# Palliative treatment of pelvic bone tumors using radioiodine (^125^I) brachytherapy

**DOI:** 10.1186/s12957-016-1050-y

**Published:** 2016-11-25

**Authors:** Chongren Wang, Zhengqi Chen, Wei Sun, Yisilamu Yasin, Chuanyin Zhang, Xiaojun Ma, Jian Chen, Jiakang Shen, Yingqi Hua, Zhengdong Cai

**Affiliations:** 1 Department of Orthopedics, Shanghai Tenth People’s Hospital, Tongji University School of Medicine, Shanghai, 200072 China; 2 Orthopedic Oncology, Shanghai General Hospital, Shanghai Jiao Tong University, Shanghai, 200080 China; 3 Shanghai Bone Tumor Institution, Shanghai, 201620 China; 4 Department of Spine Surgery, Xinjiang People’s Hospital, Urumqi, Xinjiang Autonomous Region 830001 China; 5 Department of Orthopedics, Huashan Hospital Baoshan Branch Affiliated to Fudan University, Shanghai, 200431 China

**Keywords:** Pelvic tumor, ^125^I seed, Brachytherapy, Efficacy, Safety

## Abstract

**Background:**

Complete resection of pelvic bone tumors, especially recurrent and metastatic ones, is often impossible to achieve using conventional surgery. This study aimed to assess the benefits and adverse effects of computed tomography (CT)-guided radioiodine (^125^I) brachytherapy for inoperable recurrent and metastatic bone tumors of the pelvis.

**Methods:**

This was a retrospective study of 22 patients with confirmed pelvic bone tumors (10 females and 12 males; 15–84 years; 21 with primary pelvic tumor and one with pelvic metastasis). CT-guided ^125^I brachytherapy was performed using 9–21 ^125^I seeds (radioactivity of 0.5–0.7 mCi). Seed implantation was validated by postoperative CT scanning. Complications, pain, survival, and CT-estimated tumor size were carried out to evaluate the therapeutic benefits.

**Results:**

Postoperative CT scans revealed satisfactory ^125^I seed implantation, and the radiation dose delivered to 90% of the target area (D90) was higher than the prescription dose (PD). No obvious complications were observed. Pain was reported by 19 of 22 patients, but 17 reported pain relief after implantation. Follow-up ranged 8–27 (median, 19) months. Tumor size was reduced in 11 patients within 1 month after surgery, nine patients showed no change, and tumor size increased in two patients. Finally, 1- and 2-year survival was 81.8 and 45.5%, respectively; 1- and 2-year local tumor control rates were 59.1 and 36.4%, respectively.

**Conclusions:**

^125^I seed implantation significantly reduced bone tumor size and relieved pain, with a low complication rate. These findings suggest that ^125^I brachytherapy treatment could be a useful palliative approach for pelvic bone tumor treatment.

## Background

Malignant bone tumors severely affect patient quality of life and are associated with low survival, high disability, recurrence, and mortality. In the 1970s, hemi-pelvic resection was the routine approach for patients with malignant pelvic bone tumor but only achieved a 15% 5-year survival rate. Survival was increased to 50–80% in recent years by applying comprehensive and multidisciplinary treatment strategies such as surgery combined with chemo-radiotherapy [1]. However, despite that surgery is considered as the only curative approach for bone tumors, complete resection of pelvic tumors with conventional surgery is hard to achieve due to the invasiveness of malignant bone tumors and complicated anatomic structure of the pelvis [2, 3]. The local recurrence rate of pelvic tumors is as high as 39.3% after conventional therapy [4, 5], and most patients are not fit enough to undergo a second surgery. In addition, the narrow therapeutic window of conventional chemotherapy drugs makes it difficult to increase the concentrations of drugs within the tumor tissues to improve local tumor control [6].

Brachytherapy, also known as internal radiotherapy, involves a micro-radiation source placed inside or next to a tumor. The maximum effective treatment is achieved by continuous irradiation with low-energy X-rays. A key feature of brachytherapy is localized irradiation, which significantly reduces the probability of unnecessary damage to surrounding healthy tissues [7], as observed in the treatment of various types of cancer [8]. There is no need to ensure adequate exposure of the target area or perform radical resection of the tumor tissue with conventional surgery; therefore, brachytherapy can significantly reduce surgical trauma [9]. ^125^I is a synthetic low-energy radiation source with short radiation range and a half-life of 60.1 days. It has several advantages such as long half-life, safety, reliability, and low pollution. ^125^I may cause anti-tumor effects by various mechanisms: (1) induction of tumor cell apoptosis, (2) promotion of tumor cell cycle arrest, (3) interference with tumor cell signal transduction, and (4) prevention of tumor tissue angiogenesis [10]. Grossman et al. first reported the success of ^125^I brachytherapy in the treatment of prostate cancer, with 5- and 9-year survival rates of 83 and 52%, respectively [11]. Although ^125^I brachytherapy is used as an effective treatment approach for prostate, digestive tract, lung, liver, and pancreatic cancers, it remains controversial due to local adverse effects such as skin ulceration and skin sinus formation, bladder irritation syndrome, proctitis, rectum ulceration, and prostate rectum fistulae. In addition, peripheral pulmonary emboli can occur if radiation seeds enter the systemic circulation.

Effective brachytherapy for pelvic tumors requires a high level of precision regarding the position and dose of radiation seeds in order to reduce possible adverse effects. Indeed, the complicated anatomical structure, abundant neurovascular networks, and the specific pathological type of pelvic tumor have to be taken into consideration. Probably because of the difficulties associated with pelvic operations, reports regarding ^125^I brachytherapy for the treatment of pelvic tumors are scarce.

This retrospective study aimed to assess the clinical data of 22 patients who underwent ^125^I brachytherapy between September 2009 and April 2011 for the palliative treatment of inoperable bone tumors in the pelvis. The computed tomography (CT)-guided ^125^I brachytherapy procedure was investigated, as well as its therapeutic and adverse effects.

## Methods

### Patients

This was a retrospective study of all patients with pelvic bone tumor treated with ^125^I brachytherapy by the Bone Tumor Group, Orthopedic Surgery, Shanghai Tenth People’s Hospital. Twenty-two patients (10 females, 12 males) were assessed, including six patients with chordoma; three with osteosarcoma and giant cell tumor; two with chondrosarcoma, fibrosarcoma, and melanoma; and one with leiomyosarcoma, large B cell lymphoma, intermuscular hemangioma, and neurilemmoma. Except for the patient with large B cell lymphoma, the other 21 cases had primary bone and soft tissue malignant tumors after the first conventional surgery, including the patient with melanoma with local recurrence and adjacent pelvic skeleton erosions. All diagnoses were confirmed by experienced pathologists. Patients were 15–84 years of age (median, 43 years). There was no indication of surgery for the pelvic tumors. Nineteen patients complained of various degrees of pain in the pelvis or lower extremities.

^125^I brachytherapy was carried out with the intent to narrow the resection scope of conventional surgery, reduce recurrence rate, improve the completeness of tumor resection, and preserve organ function. Inclusion criteria were as follows: (1) the tumor proliferated and locally infiltrated the surrounding organs or tissues; (2) the tumor was not easily resectable, due to large major axis length (>6 cm), extension to adjacent vasculature and nerves, and/or no clear margin; (3) isolated recurrent or metastatic bone tumor; (4) need to treat localized residual pelvic cancer after conventional surgery or external radiation therapy; (5) the patient declined conventional surgery or was unfit for conventional surgery; (6) no history of radiotherapy to the pelvis; and (7) the patient accepted brachytherapy using ^125^I seeds. The exclusion criteria were (1) active tumor bleeding, necrosis, or ulceration; (2) cancer infiltration into a large area or involving a large blood vessel; (3) non-suitability of the patient for radiotherapy, or contraindication to anesthesia; or (4) closeness of large vessels to seed implantation site.

This study was approved by the ethics committee of Shanghai Tenth People’s Hospital. The need for individual consent was waived by the committee because of the retrospective nature of the study.

### Instrument and reagents

An 18-gauge, 200-mm long puncture needle (Dr. J, Japan) was used for the delivery of the ^125^I seeds, which were purchased from the China Institute of Atomic Energy (Beijing, China). The ^125^I seeds were cylindrical (0.8 mm × 0.45 mm) with titanium capsules. The average energy was 27–35 keV, and the radioactivity ranged 0.5–0.7 mCi, with a half-life of about 60 days. Tissue penetration was about 17 mm. The initial dosing was 7 cGy/h. The TPS-200 analysis system was purchased from Hokai Medical Equipment Co. Ltd. (Zhuhai, China).

### Preoperative preparation

Each patient underwent thin-layer CT (Brilliance 64, Philips, Best, The Netherlands) 1 week before operation. The planning target volume (PTV) was designed to be a bit larger than the gross tumor volume (GTV), with 1.5-cm outward extension. A three-dimensional planning system (TPS) was used to design the optimal route around functional sensitive regions or organs. Calculations were undertaken for the required numbers of seeds and radioactivity coverage using the Hailar formula, determining the target region of the tumor, implantation site of seeds, and dosage distribution. The Hailar formula used in the current study was Hailar coefficient (Da) = (tumor length + width + height)/3; total activity of the seeds = Da × 5; number of implanted seeds = total activity/individual seed activity. The final number of seeds ranged from 9 to 21, with a median of 4.5.

### Implantation

The puncture site was determined under CT guidance. Then, the puncture needle was positioned to the predetermined site and depth after routine disinfection and anesthesia. The radioactive seeds were implanted through the needle and their position was confirmed with a CT scan. The seeds were implanted with a high density at the peripheral site of the target region and relatively sparsely at the center to achieve homogeneous radioactivity distribution. The implanted seeds were distributed in a fan-shape, with approximately 1 cm between each seed. All operations were performed by two orthopedic surgeons (Drs. Xiaojun MA and Chongren Wang), and intraoperative localization was carried out with CT-scan navigation. Radioactivity protections were used during the operation.

### Postoperative evaluation

The TPS system was used for postoperative (1 month) evaluation, and a CT scan was performed for each patient. The therapeutic effect was assessed using pain severity, tumor size, survival, and complications. Pain severity was graded according to the WHO standards [12]: grade 0, no pain; grade I, tolerable pain, no obvious effect on sleep or regular daily life; grade II, obvious intolerable pain, seriously interference with sleep, analgesics required; grade III, severe unbearable pain with disrupted sleep, associated with passive positional changes of nerves or functional autonomic nerve disorder. Pain relief outcome was classified as significantly effective (pain severity decreased by two grades or complete pain relief), effective (pain severity decreased by one grade), or invalid (no obvious pain relief or more severe pain) [13]. The KPS and VAS systems were used to assess patient symptoms, quality of life, pain, and disability, before and after treatment. Patients were reexamined by CT 2 months after surgery; the same radiologist assessed each patient before and after operation. Tumor remission was defined according to the following standards [14–16]: obvious remission (OR), tumor volume (0.5 × long axis × short axis × short axis) decreased by more than 50%; partial remission (PR), volume decreased by 25–50%; slight remission (SR), volume decreased by less than 25%; no remission (NR), no decrease or increased volume.

### Statistical analysis

Continuous variables were presented as median and interquartile ranges (IQRs) and were analyzed using the paired *t* test. *P* ≤ 0.05 was considered statistically significant. Statistical analyses were carried out using SPSS 17.0 (IBM, Armonk, NY, USA).

## Results

### Short-term evaluation after radioactive seed implantation

**Fig. 1:**
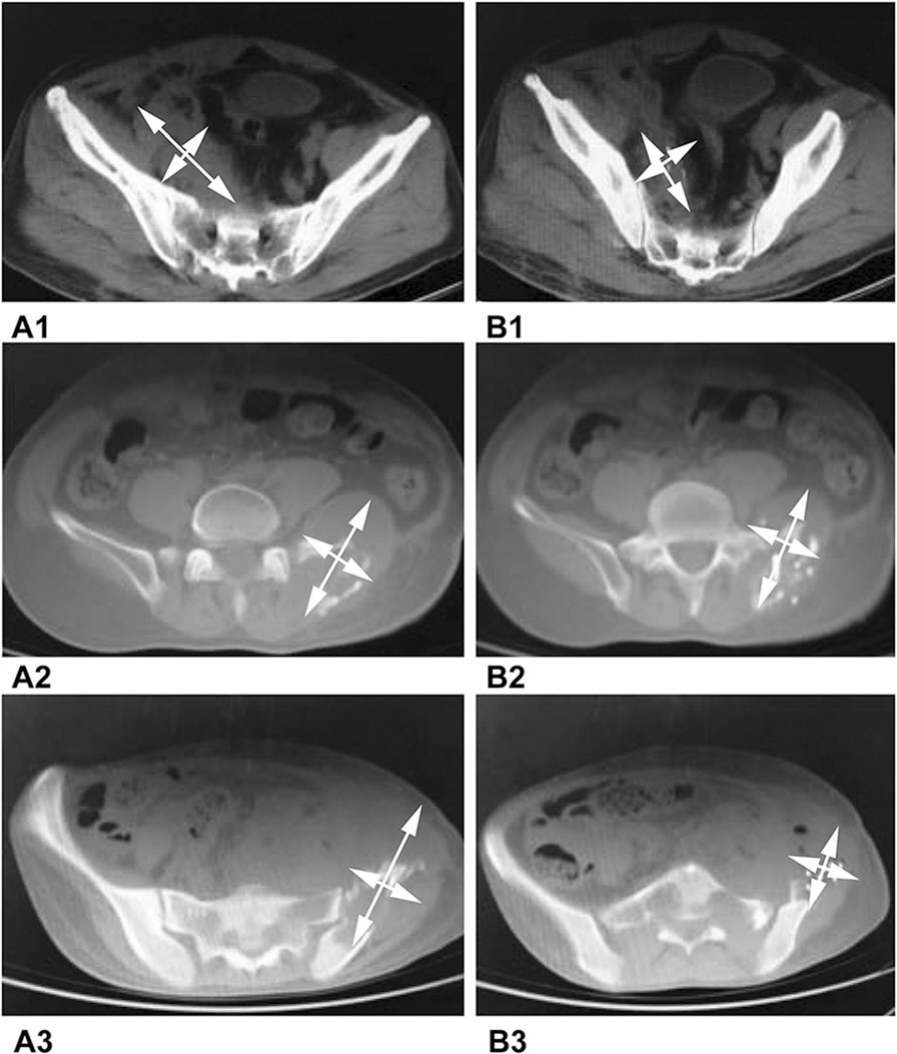
^125^I seed distribution and tumor size after brachytherapy. A1, A2, and A3 were obtained before brachytherapy; B1, B2, and B3 were recorded 2 months after brachytherapy. Seeds were evenly distributed within tumors, which were reduced in size after treatment. Obvious remission was observed in B1 and B3 (A1 diameters, 12.5 and 3.7 cm; B1 diameters, 7.3 and 2.1 cm; A3 diameters, 14.7 and 5.2 cm; B3 diameters, 5.6 and 3.3 cm). Partial remission was observed in B2 (A2 diameters, 11.2 and 6.7 cm; B2 diameters, 9.8 and 5.9 cm)

Postoperative CT and TPS evaluation revealed satisfactory ^125^I seed implantation, with a median radiation dose of 45 cGy (IQR 27.5–85.0 cGy) delivered to 90% of the target area (D90) and surpassing the prescription dose (PD) (D90 > PD). Dose inhomogeneity was no more than 20% PD. In the present study, 19 out of 22 patients showed decreased postoperative tumor volumes (Fig. 1, Table 1), and OR, PR, and SR were achieved in two, 14, and three patients, respectively.

**Table 1:** Patient characteristics and tumor size pre- and post-^125^I seed implantation in the 22 patients

				Pre-implantation tumor characteristics			Post-implantation (1 month) tumor characteristics		
Gender	Age	Pathological type	Dose (cGy)	Major axis (cm)	Minor axis (cm)	Volume (cm^3^)	Major axis (cm)	Minor axis (cm)	Volume (cm^3^)
M	34	Chordoma	80	16.7	10.2	839.2	14.5	9.1	604.65
M	58	Chordoma	30	10.1	7.9	314.7	6.6	5.2	88
M	58	Chordoma	100	22	14.4	2288	22	14.6	2346.3*
M	84	Chordoma	100	19.1	12.3	1441.7	14.8	11.8	1025.64
F	43	Chordoma	50	8	3.7	54.4	7	3.3	39.2
F	47	Chordoma	30	13.3	6.3	267.9	12.1	4.9	148.1
M	20	GCT	20	8.9	5.6	142	8	5.1	111
M	30	GCT	100	20	11.6	1350	18	9.2	768.6
F	64	GCT	60	6	4.7	67.5	5.8	4	46.4
M	75	Osteosarcoma	70	9.8	3.9	76.3	9.7	3.5	60.5
F	15	Osteosarcoma	80	18	9.6	828	14.7	8.9	582.12
F	20	Osteosarcoma	60	15.6	9.4	683.3	9.7	8.1	494.7
M	39	Chondrosarcoma	30	10.3	6.9	248	9.1	6.1	168
M	46	Chondrosarcoma	20	9.3	6.5	195.3	6.6	5.7	107.1
M	54	Fibrosarcoma	100	20.9	12.7	1681.6	19.7	12	1427.3
F	60	Fibrosarcoma	40	14.4	8.1	474.2	15.3	8.3	523.3*
F	24	Malignant melanoma	30	10.7	7.4	293.9	10	6.5	209
F	50	Malignant melanoma	30	10	7.7	368	9.5	7.5	267
M	39	B-DLCL	20	8.5	5.8	142.8	3.8	2.7	13.4
F	44	Hemangioma	10	7.4	4.6	79.5	6.5	3.9	50.7
M	61	Leiomyosarcoma	100	14.7	12.1	1070	15	13.1	1300.7*
F	16	Mesenchymal neoplasm	10	7.8	3.6	49.2	7.2	3.1	34
*n* = 22 Median (IQR)			45 (27.5–85.0)	10.5 (8.8–17.0)	7.55 (5.4–10.6)	304.3 (126.4–896.9)	9.7 (6.9–14.9)	6.3 (4.0–9.1)	188.5 (58.05–645.6)

*Tumor progression

### Pain

Regional pain was significantly alleviated in all patients after surgery. The VAS scores were significantly decreased at 1 month after implantation compared with the pre-implantation scores (*P* < 0.05; Table 2), but there was no intergroup difference at 1, 3, or 6 months after implantation (all *P* > 0.05). Nineteen patients reported pain after surgery, including one patient with grade I pain, eight with grade II, and 10 with grade III. One month after surgery, eight patients experienced significant pain relief, and nine reported effective pain relief, indicating an overall pain relief rate of 89.5%. Three months after implantation, seven patients had significant pain relief, and eight reported effective pain relief, for a pain relief rate of 78.9%. At 6 months after the implantation, four patients experienced significant pain relief, and six had effective pain relief, indicating an overall pain relief rate of 52.6%. Two patients reported complete pain relief until death. The KPS scores were significantly increased at 1 month after implantation compared with the pre-implantation scores (*P* < 0.05; Table 2). Three months after surgery, KPS scores for pain showed a further relief compared with 1 month post-implantation scores (*P* < 0.05), but the changes were not significant (*P* > 0.05) 3 months after seed implantation.

**Table 2:** Preoperative and postoperative VAS and KPS scores

Evaluation	KPS	VAS
Pre-implantation	44.5 ± 7.2	7.2 ± 1.9
1 month post-implantation*	73.2 ± 7.0	4.7 ± 1.1
3 months post-implantation*	75.0 ± 6.6	3.8 ± 1.2
6 months post-implantation*	75.9 ± 7.2	3.6 ± 1.0

*All values given as mean ± SD, *p* < 0.05 compared with the preoperative scores

### Complications

No complication associated with seed implantation (such as infection, active bleeding, nerve damage, fracture, and bone radiation necrosis) was observed. One patient experienced defecation dysfunction due to tumor progression 4 months after implantation.

### Long-term follow-up

**Fig. 2:**
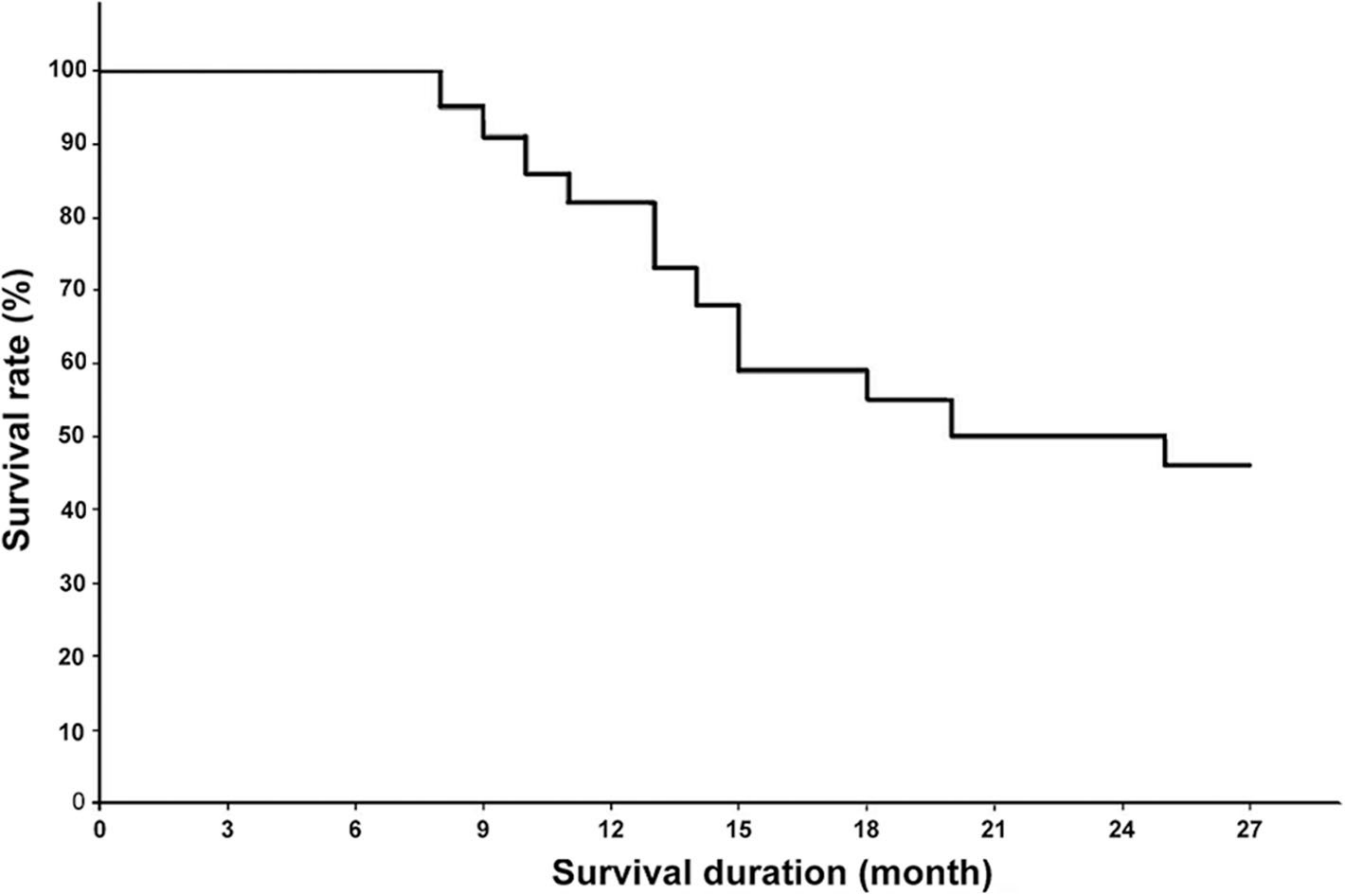
Kaplan-Meier survival curve after ^125^I brachytherapy

**Fig. 3:**
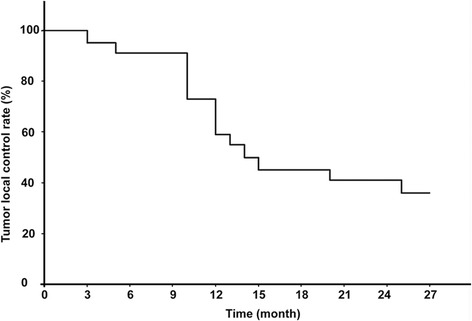
Local tumor control rates after ^125^I brachytherapy

Follow-up was 8–27 months, with a median of 19 months. The 1- and 2-year survival rates were 81.8 and 45.5%, respectively (Fig. 2). The main cause of death was multi-organ failure. The 1- and 2-year local control rates were 59.1 and 36.4%, respectively (Fig. 3).

## Discussion

Pelvis bone tumors, either primary or metastatic, often compress the adjacent soft tissues, vessels, and nerves, causing local pain, lower extremity edema, dysfunction, and paralysis. It is often impossible to achieve the radical resection of bone tumors of the pelvis because of the proximity of delicate organs and structures such as the bladder, rectum, sciatic nerve, and blood vessels [17, 18]. Although it is not very clear whether ^125^I implantation is effective against pelvis bone tumor, some reports have shown the effectiveness of external beam radiotherapy for asymptomatic or ambulatory patients with spinal cord metastases [19]. Brachytherapy has also been shown to be effective and safe in patients with spinal tumors [20–22]. Because of similar considerations in terms of delicate surrounding tissues and the risk of iatrogenic injury, the therapeutic approaches to spine and pelvis tumors share some features. Therefore, ^125^I implantation could be an important treatment modality for the quality of life of patients with pelvic bone tumors.

Radiation therapy is accepted as the first-line treatment for most patients with metastatic spinal tumors [23]. ^125^I brachytherapy was introduced for the treatment of prostate cancer in 1965 [24, 25]. The application of ^125^I brachytherapy in the treatment of spinal metastases has been reported with positive therapeutic efficacy [26–29]. Therefore, radioactive seeds could be applied for the treatment of pelvic bone tumor. The present study suggested satisfactory therapeutic effects of ^125^I brachytherapy for the palliative treatment of inoperable recurrent and metastatic pelvic tumors. Accurate implantation of radioactive seeds could be the key to achieving good therapeutic outcomes. A recent study using ablative radiation dose approach showed that CT-guided interstitial brachytherapy for recurrent anorectal cancer achieved durable tumor control, effective palliation, improved long-term survival, and minimal long-term morbidity [30]. Another study showed that high-dose brachytherapy achieved high local control rates and low toxicity in patients with pelvis tumors that were not readily accessible to other therapies [31]. Taken together, these previous studies and the present study suggest that brachytherapy could achieve high-precision treatment of tumors of the pelvic area, with minimal damage to other organs and tissues.

The low-energy radioactivity released from ^125^I seeds in a single spot is negatively associated with the squared distance to the position of the seeds; therefore, radioactivity rapidly decreases as the distance increases. The implanted seeds should be evenly distributed and sparse distribution cannot guarantee an effective dosage. Moreover, a too dense distribution is a waste of radioactive material and can cause adverse effects. CT scanning parameters such as thickness, spacing interval, and scanning direction were rigorously identical between the pre- and intra-implantation CT scans to facilitate seed implantation accuracy. Seed numbers and positions were chosen strictly according to the preoperative design, with the puncture route carefully selected to avoid injury to adjacent vessels or organs. This could be associated with the low rate of implantation-induced complications observed in the present study. The puncture angle, depth, and position of the needle tip were also adjusted under CT guidance to ensure implantation safety and accuracy. Post-implantation CT scanning was performed immediately to assess seed distribution and determine possible complications. CT scanning was also used during follow-up to assess the therapeutic effects. The present study showed that tumor size (Fig. 1, Table 1) and pain were significantly reduced, which is supported by a previous study [32]. Furthermore, it was reported that adverse side effects of brachytherapy mainly involve local adjacent organs [33–36]. Therefore, CT scanning was performed throughout the whole treatment procedure to ensure implantation accuracy and effectively avoid short- and long-term complications.

In the present study, 1- and 2-year survival rates of 81.8 and 45.5%, respectively, were observed, while the 1- and 2-year local tumor control rates were 59.1 and 36.4%, respectively. These rates are greater than those described for conventional radiotherapy, as reviewed by Lutz et al. [37]. Indeed, external beam radiotherapy for bone tumors has been shown to provide complete pain palliation for 33% of patients, but with acute and late toxicity rates of 10–37 and 1–11%, respectively [37]. In addition, median survival was around 2–4 months [37]. The present study suggests that better outcomes could be achieved with brachytherapy, as supported by previous studies. Indeed, percutaneous ^125^I seed implantation has been proposed as an alternative or retreatment option for recurrent spinal primary tumors [38]. In addition, this approach was recently described for the treatment of malignant osseous tumors [39].

A few limitations of this study should be mentioned. First, the sample size was small and from a single center, which might affect both the accuracy of our findings and their generalizability. In addition, subdividing the patients into primary and metastatic tumor subgroups was not possible because of the small sample size. Finally, a number of different types of tumors were included, which could bias the results because of different radiosensitivity. Nevertheless, our results provide a basis for the treatment of patients with malignant pelvic bone tumors not treatable by conventional methods.

## Conclusions

The results of the present study suggest that ^125^I brachytherapy could be considered a novel minimally invasive palliative treatment for inoperable recurrent and metastatic bone tumors of the pelvis. The procedure is likely to be a useful approach with few adverse effects. However, further multicenter randomized studies with a larger sample size are needed to confirm these findings.

## Abbreviations

CT: Computed tomography
GTV: Gross tumor volume
NR: No remission
OR: Obvious remission
PD: Prescription dose
PR: Partial remission
PTV: Planning target volume
TPS: Three-dimensional planning system
